# Supplementary material for: Targeted single cell expression profiling identifies integrators of sleep and metabolic state
Source: bioRxiv. 2024 Sep 27:2024.09.25.614841. Preprint. [Version 1] doi: 10.1101/2024.09.25.614841 (PMC11463630; doi:10.1101/2024.09.25.614841)
Supplement: 2 [file NIHPP2024.09.25.614841v1-supplement-2.pdf]

bioRxiv preprint doi: https://doi.org/10.1101/2024.09.25.614841; this version posted September 27, 2024. The copyright holder for this preprint (which was not certified by peer review) is the author/funder, who has granted bioRxiv a license to display the preprint in perpetuity. It is made available under aCC-BY 4.0 International license.

Table S6. Two Gene Sets Selected for GO analysis

Gene Set Category (Category)	Description	# Search Genes Found in Category	Search Genes Found in Category
FlyBase Gene Group	Biologically-revelant gene sets e.g. gene families, protein complex, shared biochemical function. http://flybase.org/lists/FBgg/	16	Adsl; AGBE; AttB; AttC; CG31075; CG32444; CG9119; Cyp28d1; deltaCOP; Got2; Inos; NimB3; Nplp2; rho; Sec61beta; Tsf1
SLIM2 GO BP	GO Biological Process subset for D.mel, for enrichment analysis . 84 terms.	15	AdSL; AGBE; AttB; AttC; CG32444; deltaCOP; Got2; inc; Inos; NimB3; Nplp2; qkr58E-3; rho; Sec61beta; Tsf1

Table S7. GO analysis results

Gene Set Category	Gene Set Name	Gene Set Size	Count Overlap Gene	log2 fold	P value	Overlaping Gene Symbols
FlyBase Gene Group	ATTACINS	4	2	8.185	1.694e-5	AttC; AttB
FlyBase Gene Group	ANTIMICROBIAL PEPTIDES	25	2	5.541	8.286e-4	AttC; AttB
SLIM2 GO BP	carbohydrate metabolic process	264	3	2.725	1.007e-2	CG32444; Inos; AGBE
SLIM2 GO BP	immune response	309	3	2.498	1.538e-2	AttB; Nplp2; AttC
FlyBase Gene Group	ISOMERASES	250	2	2.219	6.804e-2	CG32444; Inos
SLIM2 GO BP	respiratory system development	271	2	2.103	7.827e-2	rho; deltaCOP

bioRxiv preprint doi: https://doi.org/10.1101/2024.09.25.614841; this version posted September 27, 2024. The copyright holder for this preprint (which was not certified by peer review) is the author/funder, who has granted bioRxiv a license to display the preprint in perpetuity. It is made available under aCC-BY 4.0 International license.

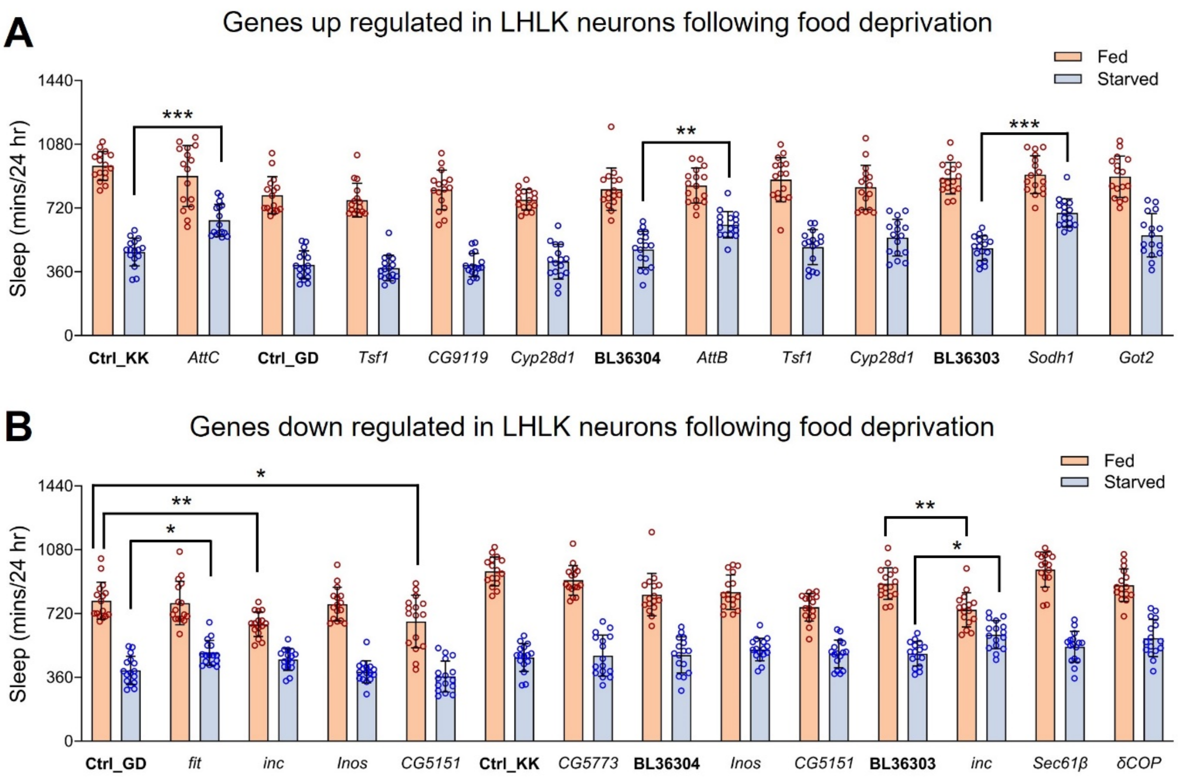

Figure Supplemental 1. Preliminary RNAi screening for genes up-regulated (A) or down-regulated (B) in LHLK neurons following 24 hours food deprivation. (**A**) Daily sleep is less reduced in *AttC*^{RNAi} (P < 0.001), *AttB*^{RNAi} (P = 0.0065), or *Sodh1*^{RNAi} (P<0.001) flies compared to the corresponding controls in response to 24hr starvation. Ctrl_KK or Ctrl_GD indicates control lines for RNA interference (RNAi) stocks from Vienna Drosophila Resource Center (VDRC). BL36303 and BL36304 are used as controls in screening TRiP stocks from Bloomington Drosophila Stock Center (BDSC). Two-way ANOVA, $F_{(12, 390)}$ = 5.188, p< 0.0001 followed with Šídák's multiple comparisons test. (B) Sleep duration is decreased in *inc*^{RNAi} (P = 0.0098) and *CG5151*^{RNAi} flies (P = 0.0307) on food and the knockdown of *fit* (P = 0.0374) or *inc* (P = 0.0465) in LHLK neurons inhibits the sleep loss induced by food deprivation. Two-way ANOVA, $F_{(13, 420)}$ = 8.480, p< 0.0001 followed with Šídák's multiple comparisons test. N = 16-20 per group. Data are mean ± SEM; *P < 0.05; **P < 0.01; ***P < 0.001.

bioRxiv preprint doi: https://doi.org/10.1101/2024.09.25.614841; this version posted September 27, 2024. The copyright holder for this preprint (which was not certified by peer review) is the author/funder, who has granted bioRxiv a license to display the preprint in perpetuity. It is made available under aCC-BY 4.0 International license.

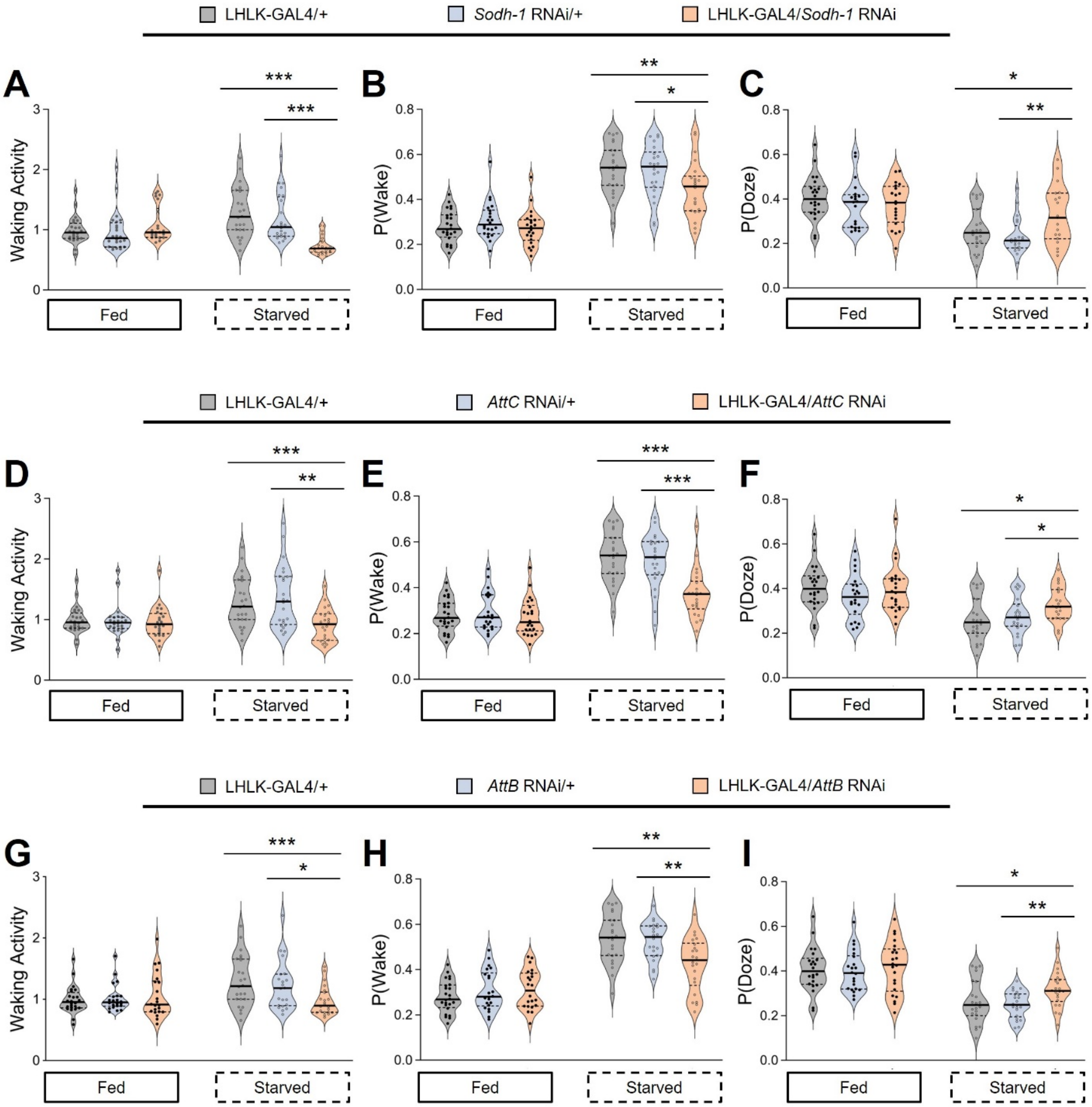

Figure Supplemental 2. Characteristics of sleep and wakefulness modulated by knockdown of *Sodh1* or *Attacins* in LHLK neurons. A,**D**,**G**) Waking activity in mutants does not differ from two control groups (light gray, light blue) under fed conditions and is reduced compared to controls under starved conditions by knockdown of *Sodh1* (**A**, P < 0.0001), *AttC* (**D**, P = 0.0009) or *AttB* (**G**, P = 0.0028) in LHLK neurons respectively. **B**,**E**,**H**) The waking propensity p(Wake) of three different knockdown flies does not differ from controls under fed conditions, and is decreased in starved flies (P = 0.0117 for LHLK-GAL4>*Sodh1*^{RNAi} in **B**, P < 0.0001 for LHLK-GAL4>*AttC*^{RNAi} in **E**, and P = 0.0017 for LHLK-GAL4>*AttB*^{RNAi} flies in **H**). **C**, **F**, **I**) The sleep propensity p(Doze) is increased in knockdown flies, which targeting *Sodh1* (**C**, P = 0.0085), *AttC* (**F**, P = 0.0115), or *AttB* (**I**, P = 0.0486) with a driver for LHLK neurons, compared to controls on agar, but not on food. Kruskall-Wallis with Dunn's post hoc. An unpaired t-test was used for

bioRxiv preprint doi: https://doi.org/10.1101/2024.09.25.614841; this version posted September 27, 2024. The copyright holder for this preprint (which was not certified by peer review) is the author/funder, who has granted bioRxiv a license to display the preprint in perpetuity. It is made available under aCC-BY 4.0 International license.

comparisons between the LHLK knockdown group and a certain genetic background control respectively. $*P < 0.05$, $**P < 0.01$ and $***P < 0.001$. Data are mean ± SEM.
